# Pump Up the Jam: Granular Media as a Quasi‐Hydraulic Fluid for Independent Control Over Isometric and Isotonic Actuation

**DOI:** 10.1002/advs.202104402

**Published:** 2022-03-27

**Authors:** Shannon E. Bakarich, Rachel Miller, Randy A. Mrozek, Maura R. O'Neill, Geoffrey A. Slipher, Robert F. Shepherd

**Affiliations:** ^1^ Autonomous Systems Division DEVCOM U.S. Army Research Laboratory Aberdeen Proving Ground MD 21005 USA; ^2^ Department of Mechanical and Aerospace Engineering Cornell University 124 Hoy Road Ithaca NY 14850 USA; ^3^ Department of Materials Science and Engineering Cornell University 214 Bard Hall Ithaca NY 14850 USA; ^4^ Weapons and Materials Research Directorate DEVCOM U.S. Army Research Laboratory Aberdeen Proving Ground MD 21005 USA

**Keywords:** fluidic elastomer actuators, granular flow, granular jamming, soft robotics, variable stiffness

## Abstract

Elastomer‐granule composites have been used to switch between soft and stiff states by applying negative pressure differentials that cause the membrane to squeeze the internal grains, inducing dilation and jamming. Applications of this phenomenon have ranged from universal gripping to adaptive mobility. Previously, the combination of this jamming phenomenon with the ability to transport grains across multiple soft actuators for shape morphing has not yet been demonstrated. In this paper, the authors demonstrate the use of hollow glass spheres as granular media that functions as a jammable “quasi‐hydraulic” fluid in a fluidic elastomeric actuator that better mimics a key featur of animal musculature: independent control over i) isotonic actuation for motion; and ii) isometric actuation for stiffening without shape change. To best implement the quasi‐hydraulic fluid, the authors design and build a fluidic device. Leveraging this combination of physical properties creates a new option for fluidic actuation that allows higher specific stiffness actuators using lower volumetric flow rates in addition to independent control over shape and stiffness. These features are showcased in a robotic catcher's mitt by stiffening the fluid in the glove's open configuration for catching, unjamming the media, then pumping additional fluid to the mitt to inflate and grasp.

## Graphical abstract

In this article, the authors implement and characterize hollow glass spheres as a quasi‐hydraulic fluid for an array of quasi‐hydraulic fluidic elastomeric actuators. These elastomer‐granular media composites are the first systems to achieve isotonic actuation (shape change) and isometric actuation (stiffness change). They define the quasi‐hydraulic as a system that combines favorable properties of pneumatic and hydraulic systems to do useful work.

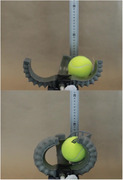

## 1 Introduction

In humans and animals, muscle drives isotonic motile actuation while isometric contraction provides the ability to resist deformation when external forces are applied on the body.^[^ ^1^, ^2^ ^]^ For example, in a dexterous grasping task of a fragile object, muscles in the human arm exhibit isotonic actuation by contracting to curl fingers of the hand around the shape of the object and then exhibit isometric actuation as the muscle modulates stiffness to bear the weight of the object. This combination of isometric and isotonic actuation enables humans and animals to move limbs both for locomotion and to bear variable loads across joints to maximize dexterity and maintain balance.^[^ ^1^, ^2^ ^]^ Using these abilities, combined with sophisticated neural architecture, animals navigate terrain and manipulate their surroundings with dexterity unmatched by humanity's machines.^[^ ^2^ ^]^

A primary objective in soft robotics is the improvement of machine dexterity and adaptability through biologically inspired design.^[^ ^3^ ^]^ One of the most popular choices of artificial muscle in soft robotics are fluidically inflated elastomer balloons, otherwise called Fluidic Elastomer Actuators (FEAs, or PneuNets).^[^ ^4^, ^5^ ^]^ In the case of FEAs, which are typically designed to bend during inflation, stiffness is coupled with level of pressurization and the induced curvature corresponds to the increased volume of gas per Equation (1).^[^ ^6^ ^]^

(1) $$\kappa =-\frac{1-\nu^{2}}{3E\pi r^{3}t}M_{N}+\frac{1-\nu^{2}}{3Et}\Delta V$$

where *κ* is curvature (a typical actuation mode), *ν* is Poisson's ratio, *E* is Young's modulus, *r* is the radius, and *t* the wall thickness of the FEA, *M_N_* is the net moment, and have Δ*V* has been substituted for Δ*P* from our prior derivation to illustrate the dependence on curvature with volume, *V*. *M_N_* is greatest when *κ* = 0 and is similarly dependent on Δ*V* per Equation (2).^[^ ^6^ ^]^

(2) $$M_{N}^{\max}=\pi r^{3}\Delta V$$

In both cases, shape change via curvature (Equation (1)), or isometric zero curvature force application (Equation (2)), the actuator must increase in volume; a twofold to threefold increase in volume is not unusual.^[^ ^7^ ^]^ However, replicating the behavior of natural muscle within a robotic system would require independent control over both stiffness and the shape or volume of an actuator. Further, an actuator with these abilities should exhibit low stiffness during shape change to reduce mechanical resistance, but also high rigidity for load bearing once the shape change is complete.^[^ ^8^ ^]^

**Figure 1:**
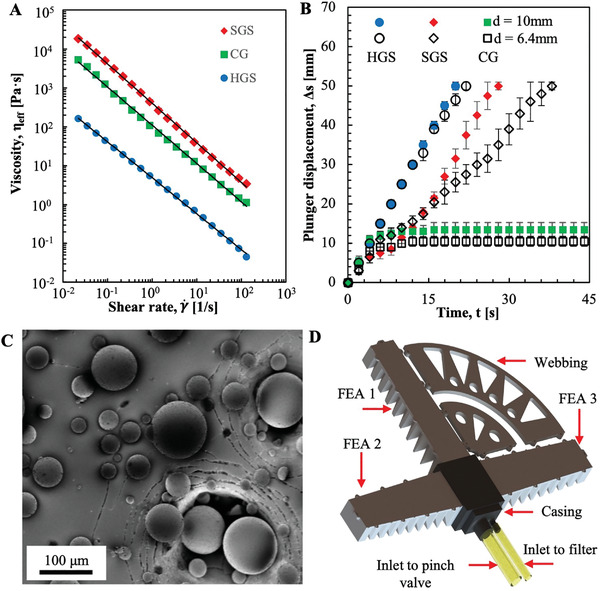
Flow behavior, selection, and demonstration of granular materials for quasi‐hydraulic fluidic elastomeric actuator: A) flow‐curves of solid glass spheres (SGSs), coffee grounds (CG), and hollow glass spheres (HGSs) measured after three pre‐shear events and fitted with the Ostwald de Waele power law model (sample size *n* = 3). B) Plot of the plunger displacement as a function of time for a pump extruding HGSs, SGSs, and CGs from 10 mL syringes through nozzle diameters of *d_i_* = 10 mm and *d_i_* = 6.4 mm (sample size *n* = 3). C) SEM images of HGSs taken after a syringe pump extruded them back and forth through a *d_i_* = 6.4 mm tapered nozzle and five pumping cycles. D) A 3D render of a robotic catching mitt made of quasi‐hydraulic fluidic elastomeric actuators (QH‐FEAs) with HGSs.

To create a lightweight system with independent control of stiffness and shape change, we looked to partner the power of hydraulic systems with the low weight and deformability of pneumatic systems by carefully selecting a “quasi‐hydraulic fluid” (QHF) and using it in elastomeric actuators. Throughout the text, we refer to this system as a “Quasi‐Hydraulic Fluidic Elastomeric Actuators” (QH‐FEAs). We used rheological techniques to predict and quantify the ability of various granules to flow easily through small orifices (**Figure** **1**A,B) and identified Hollow Glass Spheres (HGSs) as the QHF of choice when designing primarily for flowability (Figure 1C). Quasi‐hydraulic refers to the fluid's unique combination of being both incompressible like a hydraulic fluid and of very low density (*ρ* ≈ 0.15 g•cc^−1^) like fluids in a pneumatic system. We then designed a fluidic device constituted of a network of pumps and valves necessary to reversibly inflate elastomeric bladders, flow the HGSs into and out of bladders, jam the desired configurations in QH‐FEAs, and evacuate fluid from the bladders. We then characterized the highly flowable grains jamming capability in both compression and shear in 3D printed QH‐FEAs. In addition, we demonstrated the isometric and isotonic actuation capabilities of QH‐FEAs by developing a catcher's mitt that can both catch and grasp a falling object (Figure 1D).

## 2 Results

### 2.1 Engineering Parameters for Granular Media

Both inherent material properties and geometries of constituent granules strongly affect granular fluid rheological properties.^[^ ^9^, ^10^ ^]^ Therefore, we consulted the literature and considered a variety of particles that both had potential for high flowability and jamming to modulate actuator stiffness. Coffee grounds were included to compare other materials options to a standard in the literature. Solid and HGSs were examined due to their large availability in bulk quantities, low density, and high flowability. We focused on frictionless spheres (as opposed to coffee grounds) to maximize flowability, as they can move past one another without dilating.^[^ ^11^, ^12^ ^]^ However, to increase yield stress of the jammed state, granules must also exhibit “geometric friction” or resistance to flow, that is best achieved with highly frictional, non‐spherical particles that interlock when shearing past one another.^[^ ^13^ ^]^

#### 2.1.1 Quasi‐Hydraulic Granular Fluid Materials Selection

In this application, flowability is the threshold criteria for creating high frequency shape morphing and therefore was prioritized over jamming. We acknowledge that this material choice does not maximize “geometric friction” and compromises maximum actuator stiffness in favor of shape morphing capabilities. We identified two powders as potential granular fluids: i) coffee grounds (CG) because of their precedented use and favorable jamming properties;^[^ ^14^, ^15^ ^]^ and ii) glass microspheres because they are spherical and have low friction. For (ii) we used both hollow (density, *ρ* ≈ 0.15 g•cc^−1^; Fibre Glast, Inc.) and solid (*ρ* ≈ 2.5 g•cc^−1^; Cospheric, Inc.) spheres of equal diameter, *d* ≈ 70 µm, (HGSs and SGSs, respectively) to separate the effect of particle density and friction from the flow behavior.

#### 2.1.2 Quasi‐Hydraulic Granular Fluid Characterization

Rheometric data shows that the effective viscosity, *η*_eff_, of all candidate granular media increases over the number of applied shear cycles, ≈40% for HGSs, ≈30% for SGSs, and ≈60% for CSs over three cycles. We visually observed the fill level of HGSs in the reservoir cup lowered after each cycle, leading us to understand that this increase in effective viscosity is due to compaction. However, these changes in effective viscosity became smaller as the number of cycles increased. After three cycles, the HGSs ceased to compact and further shearing resulted in no further increase in effective viscosity. Therefore, since we expect our actuators to be used over several cycles, we measured the shear viscosity of the candidate powders after three pre‐shearing events (Figure 1A).

We fit flow curves for each powder with the Ostwald de Waele power law model, Equation (3):

(3) $$\eta_{\mathrm{eff}}=K{\dot{\gamma }}^{n-1}$$

where *K* is the consistency index, which can be used with viscosity measurements for comparative purposes, $\overset{\cdot }{\gamma }$, is the shear rate, and is the power law index.^[^ ^16^, ^17^ ^]^ Each of the granular fluids displayed shear thinning behavior that is characterized by an *n* < 1. HGSs have the lowest *K* (4.8 ± 0.1 Pa•s^−0.9^) which is almost two orders of magnitude less than that of the significantly denser solid glass spheres (SGS, *K* = 400 ± 30 Pa•s^−1.0^). The higher mass of the SGSs causes the powder to compact to a higher density as it is poured into the rheometer, and for granules to have greater inertia requiring higher force to accelerate the particles. The CGs have a larger *K*(110 ± 10 Pa•s^−1.0^) than the HGSs because CGs have rough surfaces that create friction, and the irregular shape of the particles enables interlocking of the grounds.

To estimate the ability of these three classes of granular material to flow through fluid networks, we used a syringe pump (plunger displacement set to 2.5 mm•s^−1^) to push granules through custom syringe nozzles. Figure 1B is a plot of the plunger's displacement, Δ*s* as a function of pumping time and *t* for each of the three granular fluids. Due to their tendency to jam, CGs do not flow appreciably for any nozzle diameter tested. SGSs flow initially, but experience a decreasing output over time for a nozzle diameter, *d_i_* = 10 mm. HGSs are the only granular system that exhibit unrestricted flow, achieved at a *d_i_* = 6.4 mm. The HGSs maintained this unrestricted flow behavior when pumped into and out of a syringe to inflate and deflate a rubber latex balloon over five cycles (Figure S1, Supporting Information). Scanning electron microscopy (SEM) images of the HGSs revealed no signs of damage to the granules from the extrusion process after five pumping cycles (Figure 1C).

We chose to use HGSs as the actuation fluid in our QH‐FEAs due to their lower *η*_eff_, lower *K*, and unrestricted flow behavior during pumping. This system demonstrated little compressibility, after pre‐shearing per our rheological protocol. Flowing 60 ± 1 mL of HGS spheres resulted in the balloon inflating to 59 ± 2 mL, a fractional compression of 1.6 ± 0.1%.

### 2.2 Quasi‐Hydraulic Fluidic Elastomeric Actuator and Fluidic Device Manufacturing and Characterization

#### 2.2.1 Fluidic Device Design

To reversibly flow and jam the HGSs in QH‐FEAs, it was necessary to design a network of pumps and valves that addressed the challenges of working with granular media. Syringe pumps are an effective tool for extruding granular fluids over short distances, but do not function well over distances needed for QH‐FEAs to do useful work. Pumping granular fluids over long distances is more challenging due to particles’ propensity for jamming in transport tubing. Granular fluids can jam when compressed between mechanical components such as the teeth of a gear pump or between the thread of a screw pump. To reduce the likelihood of jamming in the pumping apparatus, we selected a linear peristaltic pump for our fluidic device to isolate the granular fluid from the mechanism. The flexible tubing runs between a pair of rotors fitted with multiple rollers around their surfaces that compress down on the flexible tubing and squeeze the fluid along the channel.

**Figure 2:**
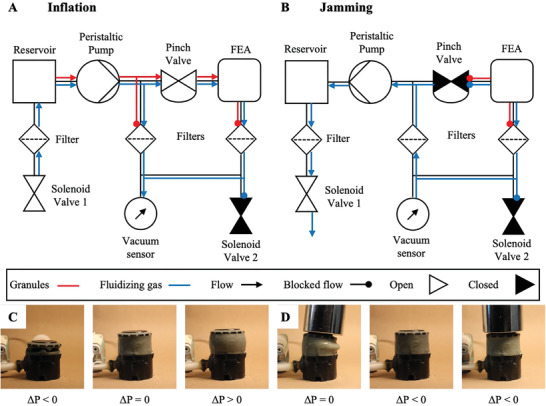
Design and operation of fluidic device. A) Flow schematic of fluidic device in configuration to inflate a QH‐FEA. B) Flow schematic of fluidic device in configuration to jam a QH‐FEA. Note that Solenoid Valve 2 is opened between states A and B to release the buildup of air pressure within the QH‐FEA. C) Photographs of cylindrical QH‐FEAs in various inflation states: (from left to right) Δ*P* < 0 evacuated, Δ*P* = 0 filled to equilibrium, Δ*P* > 0 filled beyond equilibrium. D) Photographs of filled and then jammed cylindrical QH‐FEAs demonstrating ability to bear 1 kg load. (From left to right) Δ*P* = unjammed HGSs, unable to support weight, Δ*P* < 0 induced jammed state, Δ*P* < 0 jammed state supporting weight.

We attached a 3D printed elastomeric actuator to the fluidic device and filled the reservoir with HGSs, then pumped the grains to cause actuation. The HGSs are easily fluidized, i.e., the granules are no longer geometrically constrained,^[^ ^18^ ^]^ by the surrounding atmospheric gas so no jamming occurs, even in the narrowest channels (≈6.4 mm in diameter). To control the flow direction of the HGSs, we used a network of valves and filters (**Figure** **2**A,B). We open valves to enable the flow of the fluidized grains, close the valves to block the flow and use filters to block the flow of granules while still allowing the gas to pass.

#### 2.2.2 Operation of QH‐FEA via Fluidic Device

To inflate a QH‐FEA with the fluidic device, we: 1) open the pinch valve; and 2) power the peristaltic pump to transport the fluidized mixture into the bladder. Solenoid Valve 2 is kept closed so that the pressurizing gas can inflate the QH‐FEA while the flow of HGSs backfills the expanded volume. Photographs of cylindrical QH‐FEAs inflated to various pressure states are included in Figure 2C. Once the QH‐FEA is filled, we: 3) switch off the pump; 4) close the pinch valve to constrain the HGSs within the chamber; and 5) open Solenoid Valve 2 to vent the air pressure which deflates the elastic membrane around the volume of HGSs trapped inside. We also attempted to run the fluidic controller system with Solenoid Valve 2 open during the inflation step to test whether the flow of HGSs alone would be able to expand the walls of the QH‐FEA. Under these conditions the HGSs filled the QH‐FEA but did not expand the bladder; rather, the grains began to compact down the input tubing.

To induce jamming of a QH‐FEA filled with HGSs (Figure 2B), we: 1) close Solenoid Valve 2; and 2) power either the peristaltic pump in the reverse direction or the vacuum pump. The pumps pull gas out of the chamber through a secondary line of tubing that bypasses the pinch valve. The secondary line of tubing taps into the main channel, with a filter at both ends of the tube, to prevent the granular fluid from bypassing the closed pinch valve. We embedded a pressure sensor into this secondary channel to monitor the level of vacuum in the chamber, and the jammed stiffness. Photographs of cylindrical FEAs jammed to various pressure states are included in Figure 2D. Video SV1, Supporting Information, demonstrates our controller causing a QH‐FEA to inflate with HGSs and then jam to support a load.

To de‐actuate a QH‐FEA filled with HGSs, we: 1) open the pinch valve; and 2) power the peristaltic pump in the reverse direction. The fluidized mixture flows out of the elastomeric chamber and returns to the reservoir, fully evacuating the QH‐FEA to their original state. This pump system can remove all quasi‐hydraulic fluid in multiple orientations, including when the fluid is being pumped against the force of gravity (Video SV2, Supporting Information). We imaged the granular fluid using SEM after it had been processed through the peristaltic pump (Figure S2, Supporting Information) that revealed that the rotating rollers were causing some damage to the HGSs. Rheological characterization of the crushed HGSs (Figure S3, Supporting Information) determined that this damage did not affect the flow behavior of the granules over the cycles we tested. In future work, however, due to the non‐degrading performance of granules using screw motor syringe pumps and the more compact form of the mechatronics, these systems will be used.

#### 2.2.3 QH‐FEA Characterization and Performance

**Figure 3:**
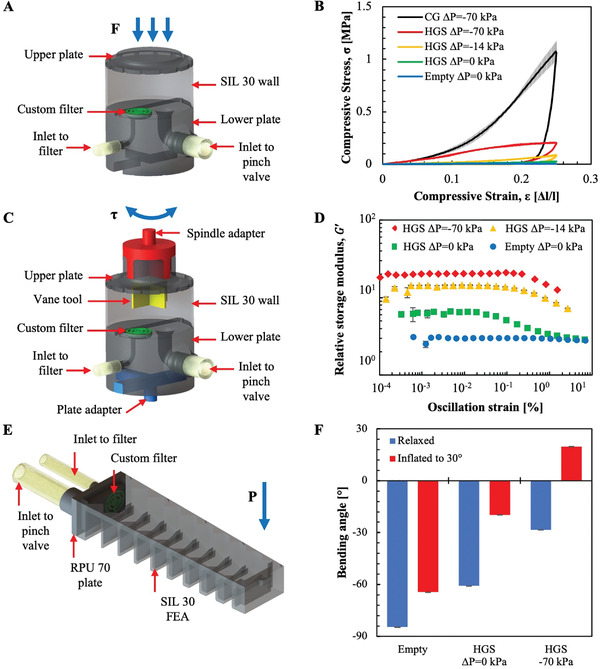
Rigidity modulation of QH‐FEA elastomer‐granular fluid composites. A) 3D render of a cylindrical QH‐FEA for compression testing. B) Stress–strain curves of cylindrical QH‐FEAs when empty (Δ*P* = 0 kPa), filled with hollow glass spheres (Δ*P* = 0, −14, and −70 kPa) and filled with coffee grounds (Δ*P* = −70 kPa) tested in compression (sample size *n* = 8). C) A 3D render of a cylindrical FEA for shear testing with adapters that couple to the rheometer. D) Torque sweeps of cylindrical QH‐FEAs when empty (Δ*P* = 0) and when filled with hollow glass spheres (Δ*P* = 0, −14, and −70 kPa) (sample size *n* = 3). E) 3D render of a bending QH‐FEA. F) Bending angle absolute deflection of bending QH‐FEA in different inflation configurations under 50 g load while empty (Δ*P* = 0 kPa for the relaxed state) and while filled with HGSs (Δ*P* = 0 kPa and −70 kPa) (sample size *n* = 3). Negative bending angles represent working upward against gravity, positive bending angles represent downward bending.

We designed and fabricated cylindrical shaped QH‐FEAs to characterize the mechanical properties of elastomer‐HGS composites under compression and shear. The cylindrical FEAs comprise hollow SIL 30 (Carbon, Inc.) cylinders (*d* = 40 mm, *l* = 20 mm, and *h* = 20 mm) capped by two circular RPU 70 (Carbon, Inc.) plates. The lower plate contains an inlet port (*d* = 6.4 mm) allowing the chamber to be pumped full of HGSs and houses a filter which allows for vacuum to be pulled on the chamber without removing HGSs (**Figure** **3**A). We demonstrate that the rigidity of the jammed state increases in compression but significantly decreases in shear.

*Compression*: The stress–strain data from the mechanical characterization of cylindrical composites is shown in Figure 3B. For compression tests, empty cylinders were placed between two compression plates. We then used our fluidic device to fill the QH‐FEA with HGSs and trigger the jamming transition in situ. To allow for the elastic membranes to maintain their printed shape but not inflate, cylinders were then filled while Solenoid Valve 2 was opened. Cylinders were compressed via materials testing machine (Zwick & Roell Z010) to a strain (*ε*  = Δ*l*/*l*_0_ ) of 0.25 and then returned to *ε* = 0 and emptied between each test.

Cylinders were tested in compression without HGSs at Δ*P* = 0. Empty cylinders exhibited a compressive modulus (*E*_c_) of 58 ± 1 kPa, compressive strength (*σ*_max_) of 6.7 ± 0.3 kPa, and energy dissipation (Γ) of 380 ± 20 J•m^−3^. However, they also exhibited buckling in compression, resulting in a negative tangent modulus (*E*_0.2_) of −6 ± 2 kPa. Filling the cylinders with HGSs increased *E*_c_ to 70 ± 10 kPa and resulted in a positive *E*_0.2_ of 130 ± 40 kPa, with a *σ*_max_ of 28 ± 7 kPa and a Γ of 1.9 ± 0.5 kJ•m^−3^. Vacuum pressure, Δ*P* = −14 kPa and Δ*P* = −70 kPa, further increased cylinder stiffness (*E*_c_ of 200 ± 30 and 600 ± 100 kPa, respectively). Cylinders filled with HGSs at Δ*P* = −14 kPa exhibited a *σ*_max_ of 90 ± 20 kPa and an Γ of 6.0 ± 1.0 kJ•m^−3^. Cylinders filled with HGSs at Δ*P* = −70 kPa exhibited a *σ*_max_ of 200 ± 10 kPa and an Γ of 22 ± 1 kJ•m^−3^.

Stiffening of these chambers is a result of the increase in packing density and increase in number of contact points between particles with the increase in pressure differential. When granular material is compressed, force chains form between contact points and resist deformation in the network of particles.^[^ ^19^ ^]^ The force chains translate compressive force radially when the cylinders are loaded, causing the granules to shear past one another and deform the elastic membrane. As both the number of contacts between particles and frictional forces acting between those contacts increase, the energy required to shear the particles past one another also increases. The jammed composites dissipate large amounts of energy because new force chains are continuously formed throughout the granular fluid each time the network structure is broken under shear. Due to the low density of the HGS media, the greatest pressure differential resulted in a specific modulus of elasticity in compression of 2400 ± 0.1 kNm·kg^−1^. Comparatively, an aqueous hydraulic fluid would have an equivalent specific modulus thirty times smaller of 80 ± 10 Nm·kg^−1^.

To draw useful conclusions regarding the mechanical performance of HGSs in our elastomer‐granular composites, it is necessary to compare their performance against CG, the standard granular fluid for jamming applications in robotics. Compression tests were also performed on cylindrical chambers filled with CGs at Δ*P* = −70 kPa. Cylinders were manually filled with CGs during the assembly process since CGs are unable to flow through the narrow tubing in the fluidic device. Cylinders filled with CGs exhibited larger *E*_c_, *σ*_max_, and Γ (900 ± 100 kPa and 8 ± 1 MPa, and 74 ± 3 kJ·m^−3^, respectively) than those filled with HGSs at the same Δ*P* (Figure 3B).

*Rheological Testing*: To quantify the effect of shear on the granules’ pressure‐induced rigidity, we designed the two ends of the cylindrical chambers to couple to the rheometer. We printed the top plate with a vane tool to ensure the bulk of the powder would be probed by an applied shear (Figure 3C). Stiffness values for these tests are reported in relative units of storage modulus, *G*′, since the shearing plates can separate during testing and the radius of the actuators fluctuate as the elastomeric chambers are filled and depressurized.

The rheological data taken from the torque sweeps is shown in Figure 3D. The empty cylinders exhibited a constant *G*′ over the entire range of applied torque, which is typical for elastic materials deformed at small strains. Cylinders filled with HGSs exhibited a 220 ± 10% increase in *G*′ over that of empty cylinders at torques below a yield point (*τ*) of 400 μn · m, which corresponds to the onset of flow among the granular media. Vacuum pressure, Δ*P* = −14 kPa and Δ*P* = −70 kPa, resulted in increases to *G*′ of 220 ± 20% and 680 ± 20%, over filled cylinders with a Δ*P* = 0, and delayed the onset of yielding to *τ* = 1 mN·m and *τ* = 2.5 mn·m, respectively.

### 2.3 Application of QH‐FEAs in Catching and Holding Robotic Mitt

We demonstrated the utility of the HGSs actuating medium in a 3D printed network of QH‐FEAs shaped to form a catcher's mitt that could stiffen in a fixed open configuration (isometric) to catch a ball and then inflate the FEAs to change shape and grip tightly around the projectile (isotonic).

We designed bending QH‐FEAs (length, *l* = 10 cm; height, *h* = 4 cm; wall thickness, *t* = 1 mm) to enhance the motion of our printed actuators (Figure 3E). These actuators, whether filled with HGS or not, weigh ≈90 g based on the volume of silicone (*ρ* ≈ 1.1 g•cc^−1^) in the solid model. Our design of the bending QH‐FEAs included a channel (width, *w* = 5 mm and height, *h* = 5 mm) that ran down the length of the actuator to aid flow of granular fluid into and out of the bladder. To quantify the stiffening effects of jamming the HGS filled actuators, we used motion tracking software to analyze the flexural rigidity of bending QH‐FEAs while inflated in different configurations.

To compare the stiffening effect of the QH‐FEA, we measured their deflection under varying conditions (Figure 3F). Negative angles denote upward deflection against gravity and positive angles indicate bending downwards with gravity. Weights were added to both inflated and uninflated QH‐FEAs in the jammed and unjammed states, as well as empty FEAs in both inflated and non‐inflated states. In the first instance, the empty actuators exhibited a bend angle of *θ* = −83 ± 2° when a point load of 50 g is applied. Filling the actuators with HGSs and applying a vacuum pressure of Δ*P* = −70 kPa resulted in a much smaller bend angle *θ* = −29 ± 2° for the same point load. We then inflated the actuators to a bending angle of *θ* = 30 ± 2° using a Δ*P* = 2.0 ± 0.2 kPa; the measured tip deflection under a 50 g point load resulted in a bending angle of *θ* = −65 ± 2°. When the QH‐FEAs were inflated with granular fluid to a bend angle of *θ* = 30 ± 2°and then a vacuum pressure of Δ*P* = −70 kPa was applied, these stiffer actuators resulted in a bending angle of *θ* = 20 ± 2° when loaded with 50 g. The only actuators to successfully hold a weight upward were the jammed QH‐FEAs, demonstrating the utility of the quasi‐hydraulic fluid and the rigidity of the jammed solid.

**Figure 4:**
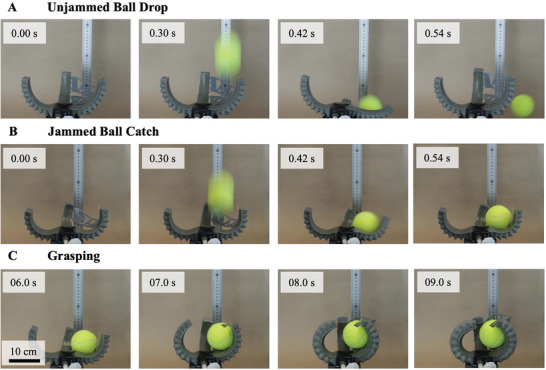
Design and control of a soft robotic catching mitt. A) Series of time lapse photographs of a tennis ball being dropped from a height of 30 cm onto an empty robotic catching mitt inflated with Δ*P* = 2.5 ± 0.2 kPa and finger deflection 50 ± 0.5 mm. B) Series of time lapse photographs of a tennis ball being dropped from a height of 30 cm onto a robotic catching mitt filled with HGSs (Δ*P* = −70 kPa) and inflated with and finger deflection 50 ± 0.5 mm. C) After time = 5 s, the pumping direction was reversed to inflate the QH‐FEAs and grasp the tennis ball. Scale bar of 10 cm is applicable to all subfigures.

Finally, we incorporated three bending QH‐FEAs into the design of a robotic catching mitt (24 cm x 14 cm x 4 cm; **Figure** **4**). Catching with a mitt is a complex task that requires both rigidity to withstand the impact of the projectile and motion for grasping. In our demonstration, the fluidic device modulated the mitt's shape and stiffness to catch a tennis ball (57 g) dropped from a height of 30 cm.

For the first catching trial, the fluidic device inflated the empty mitt with Δ*P* = 2.5 ± 0.2 kPa and finger *δ* = 50 ± 0.5 mm (Figure 4A). This low level of inflation maintains an open surface to intercept projectiles while still providing enough slack in the webbing to form a pocket to catch the ball. A series of time lapse photographs taken during this trial are shown in Figure 4A (Video SV3, Supporting Information). The pressurized mitt failed to catch the falling ball as the FEA's had insufficient stiffness to stop the projectile.

In a second trial, the fluidic device inflated the actuators in the catching mitt with HGSs to *δ* = 50 ± 0.5 mm and the vacuum pump (Δ*P* = −70 kPa) triggered the jamming transition. The mitt caught the ball on this occasion because the increased stiffness was enough to withstand the impact with a minimal deformation (Figure 4B). At time = 5 *s* the peristaltic pump was switched on to pressurize the QH‐FEAs to grasp around the ball and complete the catch (Figure 4C and Video SV4, Supporting Information).

## 3 Conclusion

We have successfully designed, characterized, and applied a granular jamming device with the capability to separate isotonic actuation (shape change) from isometric actuation (stiffness change) in our elastomer‐granule composite QH‐FEAs. Shape is controlled by changing the volume of HGSs pumped into the bladder. Stiffness is tuned by changing internal pressure of the bladder to induce jamming transitions of the particles, achieving various jamming stiffnesses by varying vacuum pressure.

The quasi‐hydraulic fluidic device we developed enables the use of HGSs as a QHF. In previous work, elastomer‐granular fluid composites have been used for variable stiffness in robotic systems,^[^ ^12^, ^13^ ^]^ and fluids were selected primarily for maximizing stiffness of the jammed state and not for flowability of the fluidized state. Coffee grounds were mainly used due to their ability to jam at high stiffness under vacuum; however, the viscosity of coffee grounds is ≈100× larger than that of the HGSs (Figure 1A). Although composites filled with HGSs dissipate ≈0.30× of the energy of the same elastomer composites filled with CGs, the combination of the flowability and jamming capability demonstrated by the HGSs makes them the more favorable jammable QHF. Due to the implementation of this fluid and device, our work is the first instance where a single mechanism has been used to control both stiffness and morphology.

The fluidic device we constructed to control our actuators allowed us to decouple stiffness and inflation. We included a peristaltic pump in our fluidic device because it could transport the HGSs through narrow channels over long distances, up to at least *l* = 1 m. We discovered, however, that a portion of the HGSs were likely crushed by the peristaltic pump. Though our mechanical and rheological data show this crushing has limited effect on the performance of the HGS media (Figure S3, Supporting Information), syringe pumps or linear motor‐based pumping (Figure 1B) would prevent this issue in future implementation. We used this quasi‐hydraulic granular fluidic device to catch a ball with a soft robotic mitt. The mitt stiffened to withstand the impact of the projectile and actuated to grip around the ball. However, this proof of concept is a demonstration of the two extremes in a pneumatic system: fully inflated and minimally inflated.

This fluidic device and elastomer‐granule composite (QH‐FEA) pushes the field of soft robotics further towards developing machines that intelligently combine softness and rigidity in a single system. The unique capability of this system to accomplish both shape change and variable rigidity lends itself well to applications such as artificial muscles or wearable assistive robotics that require these features for balance and stability. Improving the rigidity of the jammed network would enhance the load‐bearing capacity of these actuators.

Future work tailoring particles’ mechanical and surface properties in addition to further innovating the inner structure in FEA chambers will be useful for significantly enhancing network rigidity without compromising flowability and increasing the number of operation cycles. Additionally, introducing a liquid continuous state would reduce compressibility further and lower the density of the fluid, more closely mimicking a hydraulic system. In this proof‐of‐concept work, we tested the quality of the QHF after five cycles, but further experiments need to be performed in concert with these new potential design iterations to expand the design space. Future improvements in these QH‐FEAs would provide a variety of use cases such as agricultural technology and wearable assistive robotics.

## 4 Experimental Section

### Characterization of Granular Fluid Flow Properties

An Ares‐G2 rheometer (TA Instruments) coupled with a cup (*r* = 17 mm and height, *h* = 65 mm) and vane geometry (*r* = 7.5 mm) measured the viscosity of the granular fluids. The authors lowered the vane tool into the empty cup, then they poured the powder in, over the top until level with the top of the vessel. They recorded the viscosity of the granular fluids as it ramped the angular velocity of the vain tool between 0.01<*ω*< 100 rad•s^−1^ over a period of time = 60 s. They tested each cup full of material over a series of five ramp cycles with a delay of time = 10 s between each ramp cycle.

As a metric for ease of grain transport, we measured the angle of flow for each of the granular fluids with a rotating drum technique (Figure S4, Supporting Information). We 3D printed a cylinder of (*d* = 70 mm and *h* = 20 mm) in RPU 70 (Carbon Inc.) and fixed a sheet of transparent acrylic to the open end. The drum attached to a geared DC motor attached so that it could rotate around its axis. We half‐filled the drum with powder and the motor rotated it at *ω* = 0.5 Hz. We captured a series of 10 images at time = 0.5 s intervals as the drum rotated. Edge detection image analysis software (ImageJ) identified the air/powder interface and we measured the flow angle as the average slope in the center of the flow.

We investigated the flow of granular fluids through a narrow channel with a syringe pump. We printed custom syringe barrels with fixed tips so that the reservoir had an internal diameter, *d_i_* = 16.4 mm and *l* = 50 mm and the tip of syringe barrel narrowed to a *d_i_* = 10 mm and *d_i_* = 6.4 mm over *l* = 20 mm. We filled the syringe barrel with granular fluid, coupled the syringe barrel with a plunger from a disposable syringe and attached it to a linear rail guide with stepper motor (FUYU Technology Co.) with a 3D printed bracket. We placed the syringe pump on a 30° incline during operation and measured the displacement of the plunger and the mass of material extruded from the syringe as a function of time.

### Scanning Electron Microscopy

SEM images were obtained using a Gemini 500 Scanning Electron Microscope (Zeiss). Glass microspheres were mounted on carbon tape and sputtered with Iridium (Denton Desk V sputter coater) for 20 s prior to imaging.

### Fabrication of the Fluidic Device

After printing and cleaning the printed components,^[^ ^20^ ^]^ we inserted filter membranes made from 12 mm rounds of 1 Qualitive Filter Paper, Pore size 11 µm (Whatman, Inc.) and attached lengths of L/S 17 MASTERFLEX L/S Peroxide‐Cured Silicone Tubing (Cole‐Parmer, Inc.; *d_i_ =* 6.4 mm) to assemble the FEAs. We also fabricated a 3D printed T‐junction with an embedded filter attached along the length, *l* = 1 m of tubing. A peristaltic pump (MASTERFLEX L/S pump driver 7550‐50, Cole‐Parmer) attached to the silicone tubing connecting the reservoir to the FEA. A solenoid pinch valve (Cole‐Parmer) clamped onto the silicone tubing between the FEA and the T‐junction constrain the powder within the actuator. We attached a pair of syringe filters, (*d* = 25 mm, pore size 0.45 µm, PTFE, Acrosidic) to the FEA and the T‐junction which acted as backup filters and a connection point for a second length of tubing to bypass the pinch valve. A pressure sensor (PSE533‐R06, SMC pneumatics) was tapped into this second length of tubing to monitor the level of vacuum within the actuator. To control gas flow and jamming, we attached miniature solenoid valves (X‐valve; Parker‐Hannifin, Inc.) to the two ends of the fluidic system. We used an Arduino UNO microcontroller board (Arduino.cc) to control the fluidic device.

### Mechanical Characterization of Elastomer‐Granular Fluid Actuators

We measured the mechanical response of cylindrical composites in compression using a Zwick/Roell Z010 material tensile testing machine coupled with a 10 kN load cell and stainless‐steel compression plates. We placed cylindrical QH‐FEAs on the bottom compression plate. The fluidic system inflated the cylinder with HGSs and created a vacuum to jam the composite. We attached a separate vacuum pump to the fluidic device to achieve a ∆*P* = −70 kPa (Figure S5, Supporting Information). The mechanical analyzer compressed each cylinder by 5 mm at a rate of 10 mm•min^−1^ then unloaded at the same rate. We analyzed each actuator (*n* = 8) in each of the following states: i) empty (without any granular material) with valve open; ii) filled with valve open; and iii) filled with vacuum (Δ*P* = −14 and Δ*P* = −70 kPa). The fluidic device emptied and refilled the cylinders between each test. We calculated *E*_c_ as the initial slope of the stress–strain curve, *E*_0.2_ as the slope of the stress–strain curve at a strain of *ε* = 0.2, *σ*_max_ as the maximum stress of the stress–strain curve, and *G* as the area between the loading and unloading curves.

We measured the mechanical performance of cylindrical composites under shear using a DHR‐3 Rheometer with Tribo‐Rheometry Accessories (TA‐instruments). We designed and printed cylindrical tools with custom adapters that coupled with the rheometer's plate and spindle. To test the granules, we attached the empty cylinder tool to the rheometer, lowered the Tribo‐Rheometry accessory where the fluidic system filled and jammed the chamber contents in situ. The rheometer sheared the cylinders with an *ω* = 1 Hz and measured oscillation strain as torque ramped between 10^−6^ <*τ* < 10^0^ nm over a period of time = 60 s. The rheometer analyzed the cylinders in each of the following states: i) empty (without any granular material) with valve open; ii) filled with valve open; and iii) filled with vacuum (Δ*P* = −14 and Δ*P* −70 kPa). The fluidic device emptied and then refilled the cylinders between each test.

To measure the flexural rigidity of the granular FEAs at different inflation conditions, they were clamped at one end, inflated, and jammed to change deflection and stiffness. Weights were then hung from the free end in different pressurized configurations: i) empty with valve open; ii) filled with valve open; and iii) filled with vacuum (Δ*P* = −14 and Δ*P* = −70 kPa). We attached a separate vacuum pump to the fluidic device to achieve a Δ*P*  =  −70 kPa. We later actuated the tip to a deflection of 30 mm and repeated the process, closing the valve in configuration: i) for inflation (Δ*P* = 2.0 kPa). The resulting deflection from the weights were tracked using image processing software and, as before, we attached a separate vacuum pump to the actuators to reach a vacuum pressure of Δ*P* −70 kPa.

No filtering was applied to the experimental results presented in this work. Measurements are reported as the mean ± standard deviation of a number, *n*, independent experiments. In the characterization of granular materials, the flow‐curves, extrusion rates, and flow‐angle were measured in *n* = 3 independent experiments. In the characterization of QH‐FEA elastomer‐granular fluid composites, stress–strain curves of cylindrical QH‐FEAs were measured in *n* = 8 independent experiments whilst, the torque sweeps cylindrical QH‐FEAs and flexural rigidity of bending QH‐FEAs were measured in *n* = 3 independent experiments. For each of these experiments, a single FEA sample was characterized in each of the inflation states and then a new FEA sample was used for each repeat experiment.

## Conflict of Interest

The authors declare no conflict of interest.

## Supporting information

Supporting Information

Supplemental Video 1

Supplemental Video 2

Supplemental Video 3

Supplemental Video 4

## Data Availability

The data that support the findings of this study are available from the corresponding author upon reasonable request.
