# Supplementary material for: Pump Up the Jam: Granular Media as a Quasi‐Hydraulic Fluid for Independent Control Over Isometric and Isotonic Actuation
Source: Adv Sci (Weinh). 2022 Mar 27;9(15):2104402. doi: 10.1002/advs.202104402 (PMC9131430; doi:10.1002/advs.202104402)
Supplement: Supplementary file 1 — Supporting Information [file ADVS-9-2104402-s002.pdf]

ADVANCED SCIENCE
Open Access

Supporting Information

for *Adv. Sci.*, DOI 10.1002/advs.202104402

Pump Up the Jam: Granular Media as a Quasi-Hydraulic Fluid for Independent Control Over Isometric and Isotonic Actuation

Shannon E. Bakarich, Rachel Miller, Randy A. Mrozek, Maura R. O'Neill, Geoffrey A. Slipher and Robert F. Shepherd**

Supporting Information: **Pump Up the Jam: Granular Media as a Quasi-Hydraulic Fluid for Independent Control Over Isometric and Isotonic Actuation**

Shannon E. Bakarich[1,2], Rachel Miller[3], Randy A. Mrozek[4], Maura R. O'Neill[2], Geoffrey A. Slipher[1], Robert F. Shepherd[2] **

[1]Autonomous Systems Division
DEVCOM U.S. Army Research Laboratory
Aberdeen Proving Ground, MD 21005 USA

[2]Department of Mechanical and Aerospace Engineering
Cornell University
124 Hoy Road, Ithaca, NY 14850, USA

[3]Department of Materials Science and Engineering
Cornell University
214 Bard Hall Ithaca, NY 14850, USA

[4]Weapons and Materials Research Directorate
DEVCOM U.S. Army Research Laboratory
Aberdeen Proving Ground, MD 21005 USA

1. Assembly of printed components

We used a Carbon M1 3D printer to rapidly fabricate a range of structures throughout this project. The printer uses a projection stereolithography technique to draw three dimensional structures out of a vat of liquid resin. An image is projected through a transparent window on the bottom of the vat to photochemically solidify the resin. In the case of RPU 70 and SIL 30, photoirradiation produces a green state that requires a heat treatment to cure. Projection stereolithography returns short print times but can process only a single resin at a time. We printed our designs as a collection of smaller components and assembled them into composite structures following a technique previously described by the authors.[21] We washed uncured liquid resin off of printed components with isopropyl alcohol and dried them with compressed

air. We assembled our fluidic elastomer actuators (FEA) when the printed components were still in the green state to chemically bond them to one another during the heat treatment. We coated seams with a layer of liquid SIL 30 resin and solidified them with an ultraviolet light source prior to the heat treatment to enhance bonding and to ensure a seal.

2. Cyclically Syringe Pumping Hollow Glass Spheres

The main text of this paper focuses on the use of a peristaltic pump to transport granular fluids through narrow channels into and out of QH-FEAs. Here, we use a syringe pump to demonstrate an alternative mechanism to pump the hollow glass spheres (HGS) as a hydraulic fluid. These tests were conducted using the method described in "4.1.3 Syringe Pump" and "2.2.1 Fluidic Device Design," with the exceptions of: (i) Attaching a 3D printed nozzle over the cut off end of an off-the-shelf syringe barrel (Figure S1). Using an off-the-shelf syringe ensured a seal between the plunger and barrel. (ii) A rubber latex balloon was stretched over the nozzle of the syringe to act as a FEA. (iii) The syringe pump drove the plunger back and forth at its maximum speed of 1 $mm \cdot s^{-1}$ over 5 cycles and (iv) the syringe pump was sat on a level surface.

Figure S1A is a series of time lapse photographs of the HGSs inflating a balloon as they are extruded out of a syringe, and then being withdrawn back into the syringe barrel once the pumping direction was changed after time = 5 s. We visually observed the syringe filling with granular fluid, as the syringe barrel is transparent. The displacement, Δs, of the plunger as a function of time as the pump inflates and deflates the balloon with HGSs over the 5 pumping cycles is shown in Figure S1B. The plunger achieves the maximum displacement ($\Delta s = 40$ mm) after each filling event and returns to $\Delta s = 0$ mm after each emptying event for syringes with

nozzle diameters of d_i =10 mm and d_i =6.4 mm. The motor drives the syringe plunger at a constant rate throughout operation which indicates that the HGSs do not jam when extruded through the narrow channel and demonstrates that flowability of the granules is unaffected by the pumping technique.

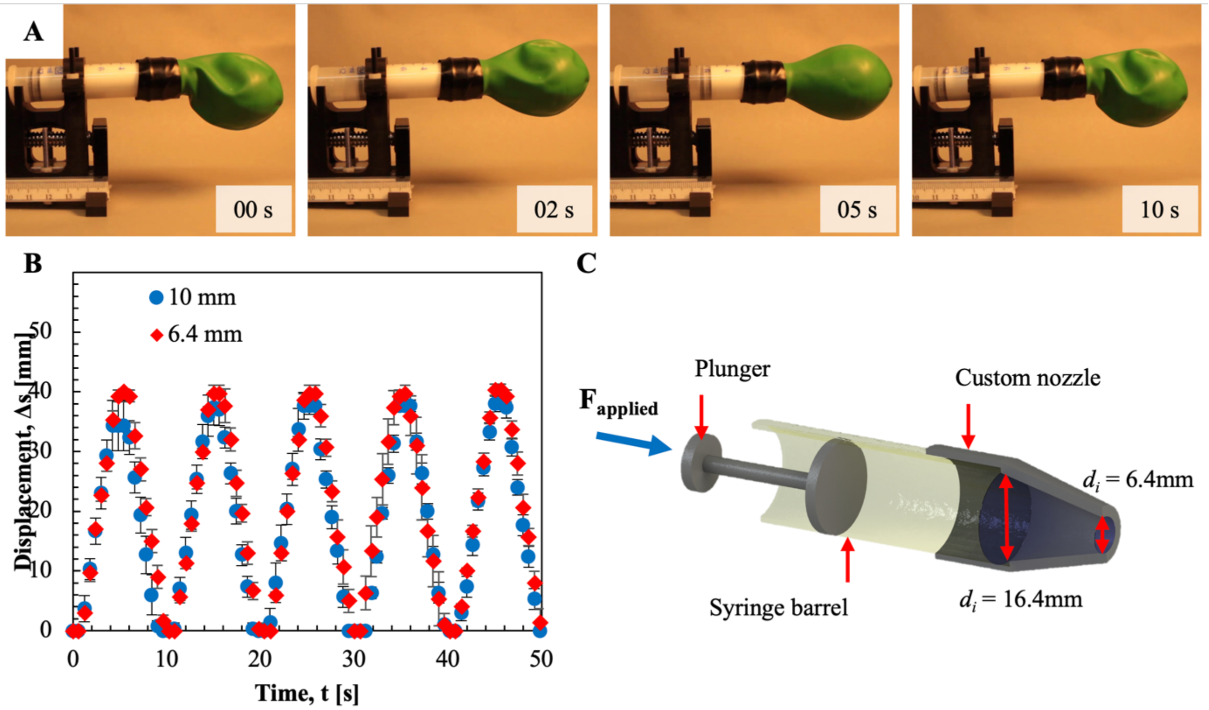

Figure S1. Cyclic pumping of hollow glass spheres with a syringe pump: (**A**) Time lapse photographs of a pump extruding HGSs through a syringe with a nozzle diameter of d_i =6.4 mm into and out of a rubber latex balloon. (**B**) Plot of the plunger displacement as a function of time for a pump extruding HGS into and out of a rubber latex balloon, through syringes with nozzle diameters of d_i =10 mm and d_i =6.4 mm (sample size n=3). **(C**) 3D model of syringe pump with custom printed nozzle.

3.0 Scanning Electron Microscopy

These tests were conducted using the method described in “4.2 Scanning Electron Microscopy”. **Figure S2A** is a SEM image of undeformed HGSs that we imaged as they were received. This image reveals spheres with a disperse range of sizes with diameters up to d~70 µm. The image also shows that there is a presence of some fragments of damaged spheres within the granular fluid. We also imaged the solid glass spheres as they were received (Fig. S2B and confirmed that the SGSs contain a high proportion of complete d=70 µm spheres.

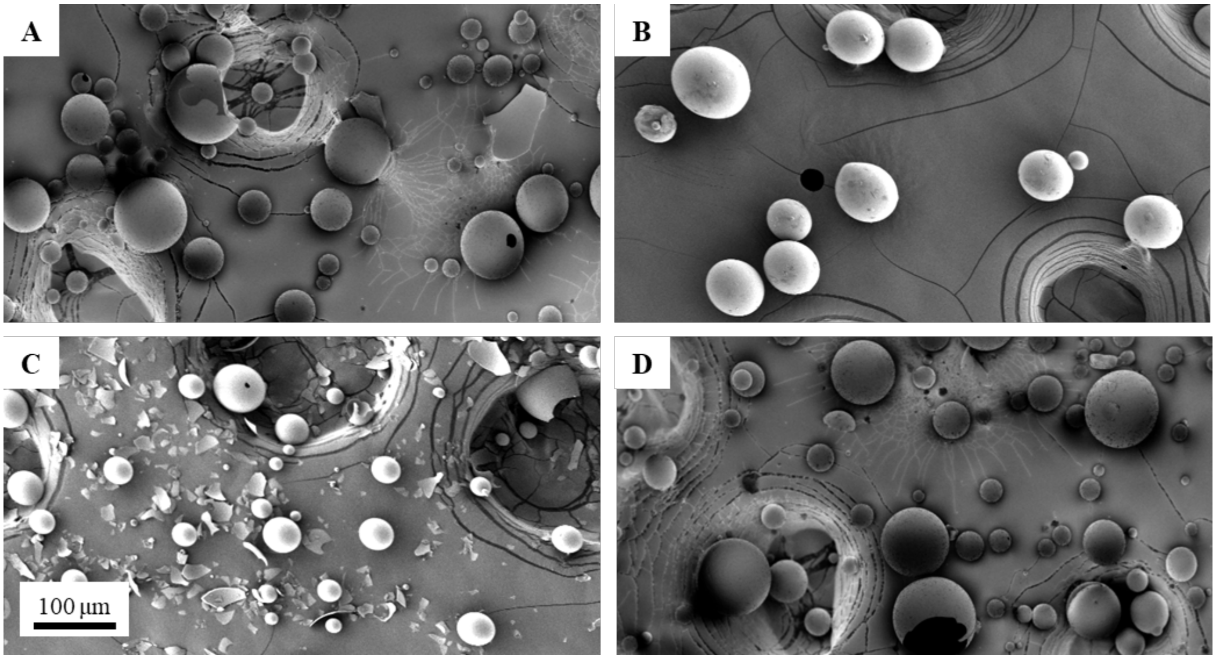

Figure S2. Scanning electron microscopy images of glass microspheres: SEM images of (**A**) Undeformed hollow glass spheres, (**B**) Undeformed solid glass spheres, (**C**) hollow glass spheres after five pumping cycles with the peristaltic pump and (**D**) hollow glass after five pumping cycles with the syringe pump.

We captured SEM images of pumped HGSs to determine the effects of each of the mechanisms on the granules. Figure S2C is a SEM image of HGSs that had been pumped back and forth

through the peristaltic pump five times prior to imaging. This image shows an absence of the largest spheres (d~70 μm) that were present in the undeformed sample and showed an increased presence of glass shards. These findings indicate that the rotating barrel mechanism crushes the hollow glass spheres during pumping. We compensated for this degradation to the HGSs in our further experiments by regularly replacing the pumped granular fluid from the reservoir with undeformed material. HGSs collected from the syringe pump after five pumping cycles (**Figure S2D**) contained a high prevalence of d~70 μm spheres and very few shards of broken granules which indicates that the syringe pump transports the fluid without crushing the spheres.

4.0 Rheology of Crushed Hollow Glass Spheres

This test was conducted using the method described in "4.1.1 Rheology." F**igure S3A** is a series of flow curves comparing the rheological behavior of undeformed and damaged HGSs. The damaged hollow glass spheres had been pushed through the peristaltic pump twice and experienced a compression event prior to being loaded into the rheometer. Damaging the spheres appeared to have minimal effect on the HGSs lowering their consistency, K to 2.0 ± 0.2 Pa·$s^{0.1}$. This small change in flow behavior does not inhibit the peristaltic pump's ability to transport the crushed HGSs.

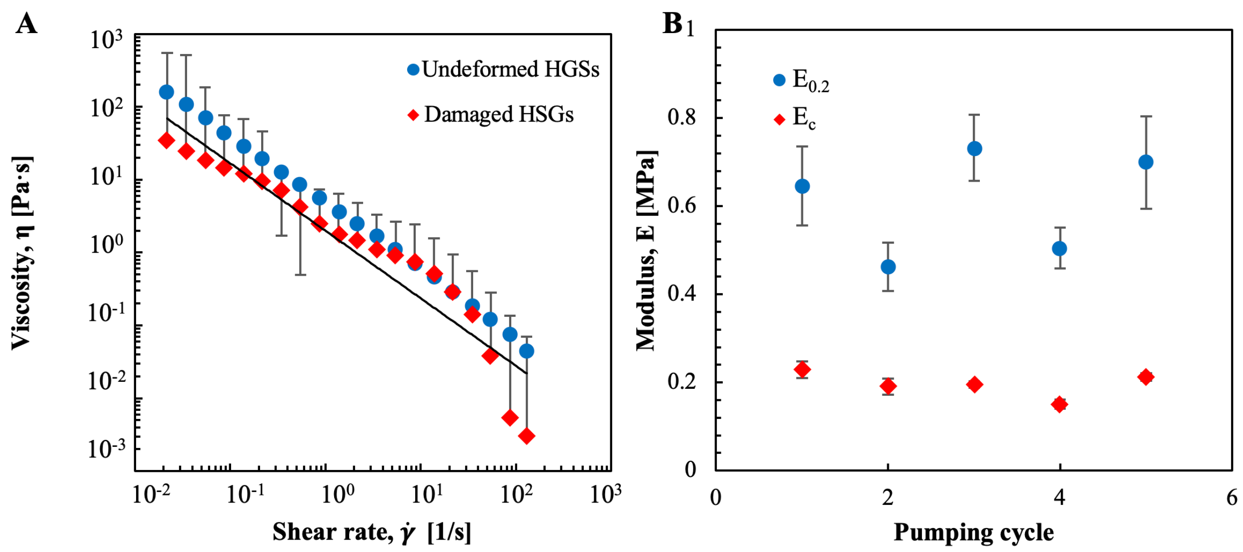

Figure S3. Cyclic use of hollow glass spheres within quasi-hydraulic fluidic elastomer actuators. (**A**) Flow-curves of undeformed and damaged hollow glass spheres measured after three pre-shear events and fitted with the Ostwald de Waele power law model (sample size n=3). (**B**) A plot of the compressive modulus, E_c and tangent modulus, $E_{0.2}$of a jammed (ΔP= -14 kPa) cylindrical fluidic elastomer actuator over a series of five pumping cycles with the same hollow glass spheres (sample size n=3).

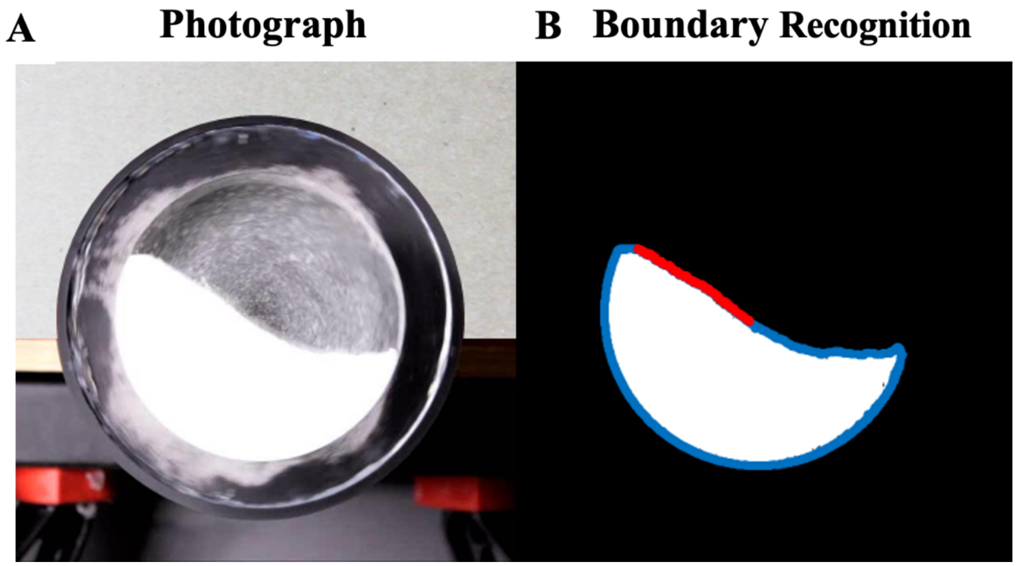

Figure S4: Boundary recognition analysis to qualitatively and quantitatively measure flowability of quasi-hydraulic fluid: (A) A photograph of hollow glass spheres in a rotating drum (ω=0.5 Hz) and **(B)** the boundary recognition analysis of that image.

5.0 Fluidic Elastomer Actuators with Cyclically Pumped Hollow Glass Spheres

This test was conducted using the method described in "2.2.3 QH-FEA Characterization and Performance" in "Compression." We measured the mechanical performance of a cylindrical QH-FEA as it was filled with the HGSs and jammed (ΔP= -14 kPa) by the peristaltic pump over a series of five cycles. We reused the same batch of HGSs throughout all cycling tests to observe the effect of crushing HGSs with the peristaltic pump on the mechanical properties of the filled QH-FEA. Figure S3B shows that the degradation to HGSs from cyclic pumping has limited effect on the compressive modulus, E_c and tangent modulus, $E_{0.2}$ of the FEA. After five pumping cycles, the jammed QH-FEA had a E_c of 210 ± 10 kPa and a $E_{0.2}$ of 700 ± 100 kPa which

represents a minor increase in stiffness compared to QH-FEAs inflated with undeformed HGSs (E_c = 200 ± 30 kPa and $E_{0.2}$ = 600 ± 100 kPa).

6.0 Attaching a Vacuum Pump to the Fluidic Device

We attached a dedicated vacuum pump (GAST, Model DOA-P704-A4) to our fluidic device which enhanced the effect of jamming on the QH-FEAs. Figure S5 is a flow schematic of the fluidic device with the attached vacuum pump in configuration to jam a QH-FEA. Attaching the vacuum pump enabled us to increase the magnitude of the pressure differential within the QH-FEAs from ΔP= -14 kPa to ΔP= -70 kPa.

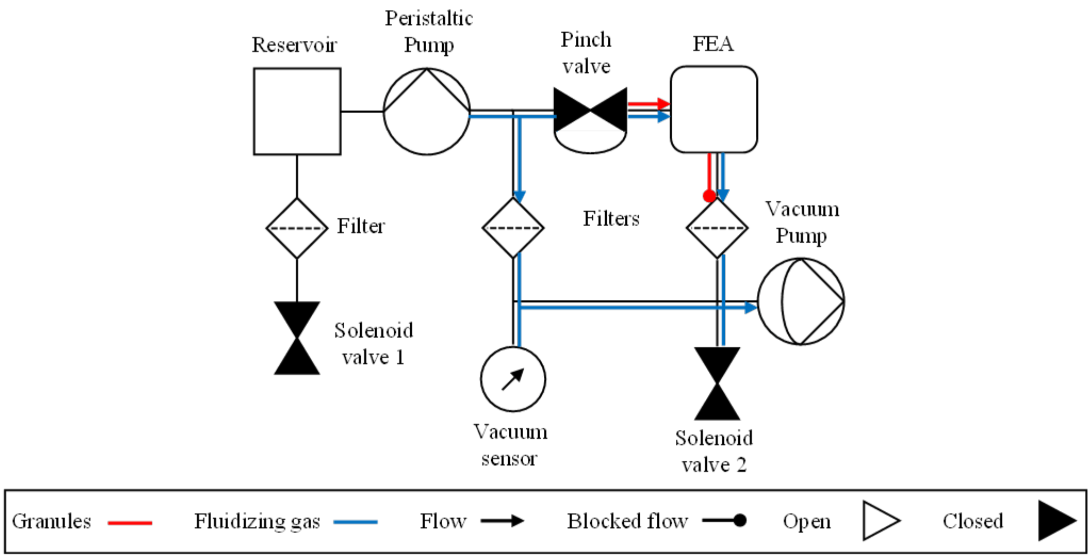

Figure S5. Design and operation of fluidic device with additional vacuum pump: Flow schematic of fluidic device with additional vacuum pump in configuration to jam a QH-FEA. The peristaltic pump is switched off whilst the fluidic device is in this configuration but the rollers in the pumping mechanism compress the flexible tubing, restricting flow in a similar manner to the pinch valve.
